# The effect of social media upward comparison on Chinese adolescent learning engagement: a moderated multiple mediation model

**DOI:** 10.1186/s40359-024-01621-z

**Published:** 2024-03-04

**Authors:** Xinjie Qi, Yi Jiang, Rong Lian

**Affiliations:** 1 Faculty of Psychology, Fujian Normal University, Fuzhou, China; 2 Fujian Chuanzheng Communications College, Fuzhou, China

**Keywords:** Teenagers, Social Media Upward comparison, Learning Engagement, Sense of Agency, Growth mindset, Positive - negative emotions

## Abstract

To investigate the mechanisms and boundary conditions of social media upward comparison on adolescents’ learning engagement, this study utilized two separate approaches. In research 1, a convenience sampling method was employed to conduct a questionnaire survey among 609 adolescents. The aim was to explore the influence of social media’s upward comparative tendency on learning engagement. In research 2, real social media platforms were utilized to select short videos as experimental materials. These materials were used to manipulate instantaneous social media upward comparison and examine its impact on learning engagement. The research 1’s result indicated that the mediating effect of the sense of agency was significant, as well as the moderating effect of growth mindset. However, the mediating effect of positive-negative emotions was found to be insignificant.Regarding instantaneous social media upward comparison and learning engagement, positive and negative emotions were found to play a complete mediating role. However, the mediating role of agency and the moderating role of growth mindset were not significant.This study offers practical insights for educators, enabling them to develop a correct understanding and effectively regulate adolescents’ use of social media.

**Supplementary Information:**

The online version contains supplementary material available at 10.1186/s40359-024-01621-z.

## Introduction

According to the 51st Statistical Report on Internet Development in China released by the China Internet Network Information Center (CNNIC), as of December 2022, the number of internet users in China reached 1.067 billion, with adolescents being the main users of social media. Currently, research has demonstrated the influence of social media usage on adolescents’ interpersonal relationships [1], self-perception [2], and negative emotions such as depression [3]. While existing longitudinal research suggests that excessive engagement with social media may diminish the extent of academic engagement among university students [4], there is no clear consensus regarding the effects that regular social media usage has on the learning engagement of adolescents. Further investigation is required to elucidate the nuanced implications of social media interaction within this younger demographic.

Chinese traditional culture emphasizes the significant impact of learning outcomes on the quality of future life. At the same time, Chinese adolescents are affected by the elimination system of the middle school and college entrance examinations, which leads them to place more importance on the results of learning compared to adolescents from other countries. Therefore, they also place more emphasis on the investment in learning. Learning engagement is a sustained state characterized by individuals exhibiting positive emotions during the learning process [5]. It serves as a crucial indicator reflecting students’ daily learning status [6]. Not only is it closely related to individuals’ happiness and academic achievement, but it also promotes students’ cognitive and mental well-being, addressing major educational issues like academic burnout [7].

According to the ecological systems theory, learning engagement among adolescents is the result of the interaction between personal and environmental factors [8]. Previous research has shown that family factors, such as parenting styles [9], classroom and school atmosphere [10], and peer interpersonal relationships [11], are significant external environmental factors that predict levels of learning engagement. However, the impact of social media use, which is closely related to modern lifestyles, on adolescents’ learning engagement has been overlooked. Previous perspectives [12–14] suggest that social media use detracts from academic engagement by monopolizing time that might otherwise be devoted to scholarly activities. On the other hand, the compensation theory proposes that social media can effectively fulfill unmet social needs among adolescents [15], enhance their self-efficacy [16], and subsequently improve their learning engagement [17], Even a research shows that when higher education students use social media’s knowledge-sharing capabilities, their academic performance improves [18]. As a result, investigating the correlation between social media use and adolescent learning engagement holds substantial practical significance.

## Literature review

### The relationship between social media upward comparison and learning engagement

With the widespread popularity of the Internet, smartphones, and other convenient mobile devices, people have become accustomed to sharing various aspects of their lives on social media platforms. A survey targeting the 27 EU countries revealed that 80% of individuals aged 16–24 use social media [19]. In China, the proportion of adolescent users is also the highest on several social media platforms that are commonly used (WeChat Moments, QQ Space, and Xiaohongshu) [15].Thus, teenagers are the major users of social media.

Whether actively or passively browsing through social media content, individuals naturally engage in social comparisons [20]. Consequently, social media has emerged as the primary platform for individuals to engage in such comparisons [21]. Social comparison is a universally observed social psychological phenomenon [22], where in individuals frequently engage in self-evaluation by comparing themselves to others. For adolescents, who are still in the process of constructing their self-concept and have relatively simple social support networks, comparing themselves to others becomes an important means of self-recognition and positioning [23]. In addition to comparing themselves with classmates and friends in their immediate surroundings, teenagers now increasingly compare themselves to their peers on social media platforms due to the widespread use of mobile devices [24].

However, on social media, individuals often display items, abilities, and lifestyles that ordinary people lack but aspire to possess due to economic reasons for attracting attention. This easily triggers upward comparisons among teenagers when they browse through such information [25]. Numerous studies have demonstrated that engaging in upward comparisons on social media can lead to reduced self-esteem [26], as well as feelings of jealousy, depression [3], anxiety [27, 28],body image perception [1]. A meta-analysis further confirmed these significant negative effects [29]. It is worth noting that high levels of self-esteem and positive emotions have been shown to positively predict individuals’ level of learning engagement [8]. Meanwhile, when adolescents engage in upward comparisons on social media, they often need to allocate their limited cognitive resources to cope with the negative emotions triggered by such comparisons and restore their diminished self-esteem [19]. This process could potentially hinder their ability to regulate their learning motivation and actively engage in learning activities [30]. It is worth noting that the aforementioned studies utilize self-reported questionnaires to measure upward comparison, which actually assess individual behavioral tendencies. As a result, we formulate *hypothesis 1: the upward comparison tendency on social media may reduce adolescents’ learning engagement (H1).*

Conversely, from the cognitive self-regulation perspective [31], displaying positive learning outcomes can enhance adolescents’ mental representation of learning behaviors and improve their intention to engage in learning.The emotional contagion theory of digital media proposes that people can be affected by the emotions conveyed in online content. When individuals view images, text, or short videos with positive or negative emotions on social media, they may unconsciously mimic those emotions [32]. This automatic imitation, influenced by physiological feedback, causes viewers to feel the same emotions as portrayed in the content, leading to either a more positive or negative emotional state [33]. Experimental studies have shown that positive content on social media platforms can generate positive emotions among users and promote constructive behaviors beneficial to personal development, such as learning and fitness [34, 35]. Choi and Kim found that the positive emotions generated from browsing influencers’ Instagram could counteract the negative effects of upward comparison [36], indicating that upward comparison on social media may not necessarily diminish subjective well-being. The conclusions from the mentioned studies are based on immediate behaviors observed in experiments. Apart from traditional images and text on social media, the popularity of short videos has made it easier for people to get into a focused state (flow) while browsing [37]. This state of flow can bring pleasure and fulfillment. Additionally, short videos quickly convey emotions, and in the context of quick comparisons, emotional impact on learning engagement is considered more significant than cognitive changes. Also, content triggering upward comparison on social media often contains positive emotions. Hence, we put forward the hypothesis 2 that *instantaneous social media upward comparison could foster learning engagement (H2).*

Obviously, it remains unclear how social media upward comparison impacts adolescents’ learning engagement, whether there are differences between long-term behavioral tendencies and short-term states, and what mechanisms underlie these influences. To fully understand the impact of social media upward comparison on individuals, it is necessary to consider both behavioral tendencies and instant states of social media upward comparison.

### How social media upward comparison impacts learning engagement

#### Cognitive path

##### Sense of agency

Intrinsic motivation and self-regulation abilities have been shown to positively predict levels of learning engagement [38]. Some studies [25, 26, 28] have shown that upward social comparison could increase feelings of relative deprivation and subsequently diminish individuals’ intrinsic motivation through behavioral experiments. Liu and colleagues also discovered a positive relationship between relative deprivation and aggressive behavior mediated by a higher sense of agency [39]. These findings suggest that upward comparison may reduce intrinsic motivation and self-regulation by weakening the sense of agency. The sense of agency refers to an individual’s subjective experience of controlling their own actions and the external environment generated during active behavior, which forms the basis of self-regulation [40].From the perspective of expected value theory, it is evident that the interplay of subjective valuation, anticipation of success, and the perception of internal control are key determinants shaping the contemplation, strategizing, and implementation of any actionable endeavor [41]. Luo, Wang, and Zhou also demonstrated the sense of agency can enhance individuals’ cognitive performance, which serves as a crucial foundation for learning engagement [42]. This suggests that the sense of agency positively predicts learning engagement. Furthermore, in accordance with reinforcement learning theory, the development of a sense of agency is intricately linked to the process of learning from the correlations between one’s behavioral actions and the resulting environmental feedback. This connection underscores the significance of experience in shaping an individual’s belief in their ability to influence outcomes through their choices and actions [43]. Therefore, The disparity between others and oneself generated by comparison on social media accelerates the instability in the construction of self-concept among adolescents. This can lead to a learned helplessness cognition that no matter how much effort they exert, they cannot achieve the same level as the individuals portrayed on social media. Consequently,We propose the following hypothesis 3 that *sense of agency plays a mediating role in the relations between social media upward comparison and learning engagement(H3).*

#### Affective path

If changes in the sense of agency and growth mindset represent the cognitive aspect of social media upward comparison mechanisms that influence adolescents’ learning engagement, then positive-negative emotional experiences represent the emotional aspect, often referred to as the “hot” part. According to the emotional contagion theory, emotional information is automatically and unconsciously transmitted between individuals, leading to a synchronization of emotions between the sender and receiver [32]. Kramer, Guillory, and Hancock demonstrated that emotional contagion can also occur in virtual social situations with indirect social interactions and non-verbal cues [44]. This provides a new perspective for understanding the impact of social media upward comparisons on individuals.

##### The positive emotion

On social media platforms, people tend to display content that highlights their superiority and positivity, which inherently contains positive emotional components. When viewers consume such content, they are automatically and unconsciously infected by the positive emotions portrayed in it, leading to feelings of emotional pleasure [36]. Pleasant emotions(like happy, joys, hopeful)have been found to enhance self-efficacy [9], thereby increasing levels of learning engagement [10].Additionally, from the perspective of embodied emotion, when observers mimic and generate an emotional experience consistent with the content they browse, such embodied behavior feedback to the brain, enhancing positive emotional experience again through changes in internal body states, such as heart rate and breathing patterns [45]. Positive emotions can lead to deeper and steadier breathing in individuals, enhancing their sense of relaxation. Furthermore, the increased heart rate variability (HRV) associated with positive emotions indicates better adaptability and resilience in the body [46], which are undoubtedly beneficial for adolescents’ engagement in learning.

##### The negative emotion

Although the current study primarily focuses on social media content that triggers upward social comparisons among individuals, which mostly showcases one’s positive aspects, it is less likely to induce negative emotions in viewers through emotional contagion. The gap created by upward comparison may lead to negative emotions such as disappointment, helplessness, depression, and anxiety in adolescents. Similarly, based on the embodied emotion theory [46], negative emotions can cause breathing to become faster and shallower, and HRV may decrease, exacerbating the individual’s feelings of tension or unease. Meanwhile, the process of regulating these negative emotions consumes limited cognitive resources [47], leading to reduced self-regulation [48],which is the core ability of learning engagement [49].

From this perspective, the positive emotions produced through emotional contagion can enhance adolescents’ engagement in learning, while the negative emotions resulting from the disparity in comparisons may weaken their engagement. Consequently, we cannot formulate specific hypotheses about the outcomes of emotional pathway changes at present; we can only generally hypothesize that *emotional experiences act as mediators between upward social media comparisons and learning engagement (H4)*. However, we will separately examine the pathways of positive and negative emotions.

#### Growth mindset

Although traditional social comparison theories suggest that upward comparisons hinder individuals’ constructive behaviors. Diel and colleagues have discovered that upward comparisons in areas that individuals regard as important or where they excel can inspire a motivation to change, which in turn can lead to convergence effects [50]. Additionally, social learning theory suggests that observing others’ success helps students acquire knowledge and skills through social learning and enhances their self-efficacy [51]. The motivation enhancement resulting from upward comparisons can actually promote individuals’ learning behaviors. In a study conducted by Song and his colleagues in the workplace, upward comparisons were found to stimulate positive emotions and constructive behaviors such as learning and self-improvement [52]. An important moderating factor in this relationship was learning goal orientation. Individuals with a learning goal orientation focus on their own changes and progress in the past, present, and future.

A prerequisite for learning goal-oriented behavior is a growth mindset [53]. Individuals with a growth mindset believe that intelligence and abilities can be improved through effort and training [54]. They are also more inclined to face stress and failure with a developmental and positive mindset [55]. An intervention study conducted nationwide on academically under performing ninth-grade students in the United States also demonstrated that even short-term (less than one hour) online training in a growth mindset significantly improved students’ academic performance and overall college enrollment rates [53]. This indicates that individuals with a growth mindset, whether adolescents or adults, tend to perform better in academic performance [56], cognitive functioning [57], and subjective well-being [58]. As previously mentioned [50], when the content of upward comparison is something that the individual values, such upward comparison can stimulate the individual’s behavioral motivation and exert a facilitating effect. Chinese adolescents generally place a high value on academic achievement. Therefore, when they browse content showcasing their peers’ excellent academic performance, those with a growth mindset may be more easily inspired to enhance their motivation to study. Furthermore, the essence of a growth mindset is future-oriented, and individuals with a growth mindset can gain more psychological resources by looking to the future [59]. According to the cognitive appraisal model of stress [60], individuals with abundant psychological resources are less likely to perceive upward comparisons on social media as stressful events, thereby mitigating the negative effects of upward comparisons. Consequently, we posit a hypothesis 5 that *growth mindset plays a mediating role in the relations between social media upward comparison and learning engagement.(H5)*.

### The current study

**Fig. 1:**
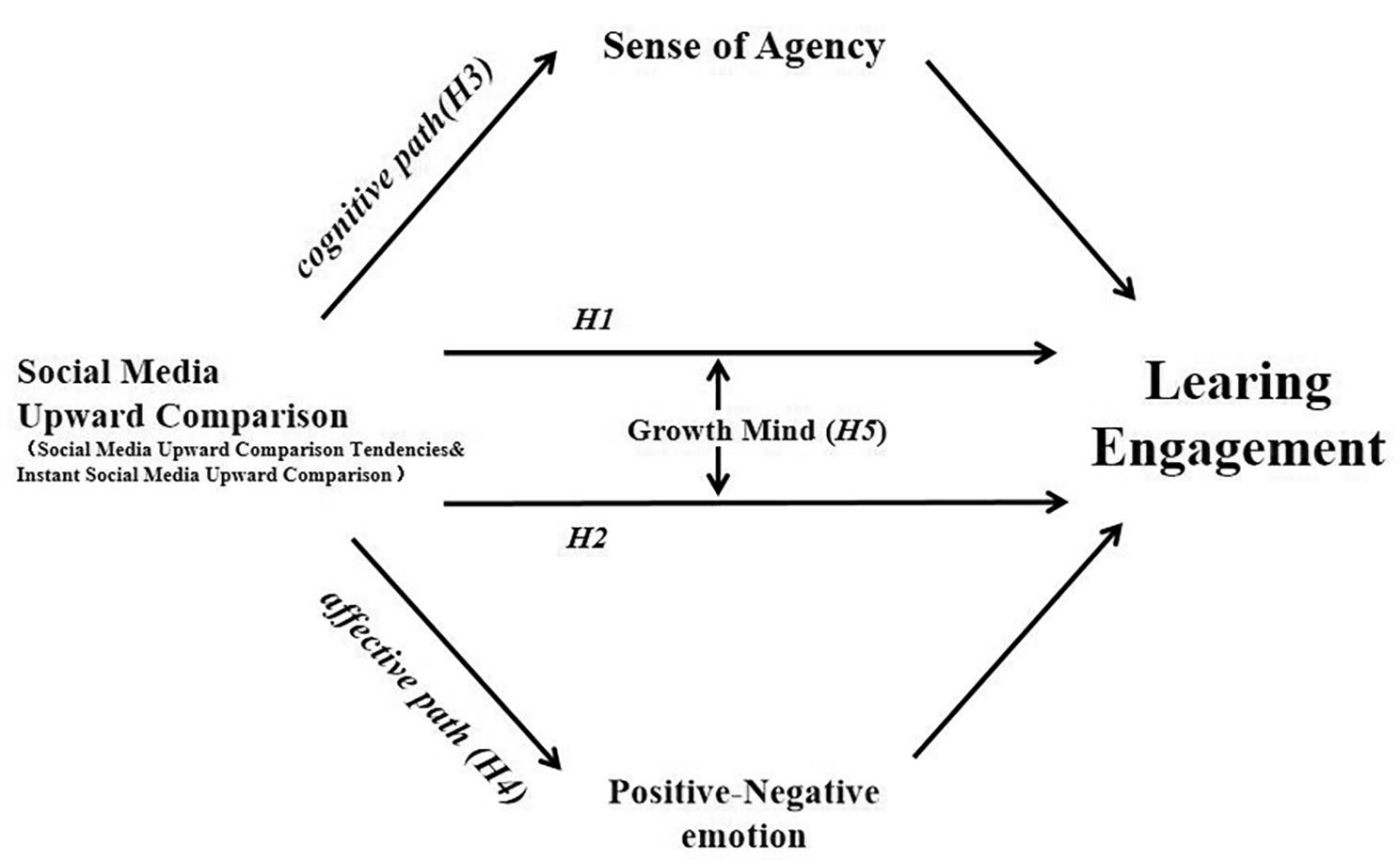
Theoretical hypothesis model

Due to a dearth of comprehensive research investigating the impact of social media upward comparison on learning engagement, further investigation is warranted. This study seeks to explore how both long-term behavioral tendencies and instantaneous states of social media upward comparison affect adolescents’ learning engagement through questionnaire surveys(Research 1) and experimental designs(Research 2). Drawing upon theories related to social comparison and emotional contagion, we hypothesis that the upward comparison tendencies on social media significantly negatively predicts learning engagement(H1), while the instantaneous upward comparison on social media significantly positively predicts learning engagement(H2). In cognitive pathway, the sense of agency serves as a mediating factor between upward social media comparison and learning engagement(H3), whereby engaging in upward social media comparison diminishes learning engagement by undermining the sense of agency. In affective pathway, emotional experience plays a mediating role in the relationship between social media upward comparison(tendency and instantaneous state) and learning engagement(H4). Specifically, social media upward comparison promotes learning engagement by enhancing positive emotional experiences. At least, we hypothesis that growth mindset moderates the relationship between upward comparisons on social media and learning engagement(H5). The theoretical hypothesis model is illustrated in Fig. 1.

### Research 1

Research 1 utilized a questionnaire to examine the influence of upward social comparison tendency on learning engagement among adolescents on social media.

## Method

### Participants

Based on the $n = \frac{{{z^2} \times p \times (1 - p)}}{{{E^2}}}$ calculation for sample size, with a 95% confidence level where the *Z* = 1.96, *p* = 0.5, and *E* = 0.05, the calculated sample size is approximately 385. A convenience sampling method was utilized to select 613 students from a public middle school in Fujian Province. With the guidance of graduate students majoring in psychology, participants completed the questionnaire during a 15-minute session within their class time. After excluding duplicate and incomplete responses (less than 2 min of answering time), a total of 609 valid questionnaires were obtained, including 303 from boys and 306 from girls(*M*(age) = 15.18, *SD* = 0.59). The distribution across grade levels was as follows: Seventh Grade(*n* = 235), Eighth Grade (*n* = 214), and Tenth Grade (*n* = 160).

### Measures

#### Upward comparative tendency of social media

To measure upward comparative tendency on social media, the upward comparative subscale from the Comparative Propensity Scalewas employed. The scale has been validated to have good reliability and validity among Chinese adolescent groups [61]. For the purpose of this study, the comparison range in the questionnaire was modified to “in social media”. The questionnaire consisted of six items rated on a five-point scale ranging from *strongly disagree* to *strongly agree*. Higher scores indicated a higher frequency of engaging in upward comparison on social media platforms. In this research, Cronbach’s α coefficient for internal consistency reliability was calculated as 0.86.

#### Sense of agency

The Chinese version of the Sense of Agency Scale [62]was utilized in this study. The scale consists of nine items that are divided into two dimensions: positive and negative sense of agency. Participants rated each item on a seven-point scale, with 1 indicating *strongly disagree* and 7 indicating *strongly agree*. A higher score reflects a greater sense of agency. The internal consistency reliability of the scale in this study was acceptable, with a Cronbach’s α coefficient of 0.91.

#### Learning engagement

The Chinese version of the Learning Engagement Scale [63] was employed to assess learning engagement. The scale consists of 17 items. Participants rated each item on a five-point scale, with higher scores indicating greater learning effort. The internal consistency reliability of the scale in this study was high, with a Cronbach’s α coefficient of 0.95.

#### Positive-Negative emotion

The Chinese version of the Positive-Negative Emotion Scale (PANAS) [64], was used to assess participants’ emotional states during the study period. The PANAS includes two subscales: positive emotion and negative emotion. Participants rated their emotional experiences using a five-point Likert scale for each adjective listed under positive emotions and negative emotions, respectively. Higher scores on each subscale indicate a stronger experience of the corresponding emotion. The internal consistency reliability of the scale in this study was good, with a Cronbach’s α coefficient of 0.88 and 0.89 in positive and negative emotion respectively.

#### Growth mindset

The Growth Mindset Pattern Scale was employed to measure participants’ inclination towards a growth mindset, which has been validated to have good reliability and validity among Chinese adolescent [65]. The scale consists of six items. Participants rated each item on a six-point scale, with 1 representing *completely disagree* and 6 representing *completely agree.* A higher score indicates a greater likelihood of having a growth mindset. The internal consistency reliability of the scale in this study was satisfactory, with a Cronbach’s α coefficient of 0.89.

## Results

### Common method deviation test

To examine common method bias, the Harman single-factor test was conducted. Among the 58 items used in the study, seven common factors with eigenvalues greater than 1 were extracted. The first common factor accounted for 26.67% of the variance, which was less than the critical threshold of 40%. These results indicate that there is no significant common method bias present in this study. Descriptive statistics, including means and standard deviations, as well as correlation coefficients among variables, are presented in Table 1.

**Table 1:** Descriptive statistics and correlation coefficient matrix of variables in Study 1 (*n* = 609)

	M ± SD	1	2	3	4	5	6
1.Social Media Upward Comparative Tendencies	19.01 ± 9.22						
2.Sense of Agency	40.10 ± 9.08	-0.37**					
3.Positive Emotion	24.16 ± 14.29	0.06	0.03				
4.Negative Emotion	24.62 ± 14.63	-0.05	0.09*	-0.01			
5.Growth Mindset	20.61 ± 4.54	0.09*	0.11**	0.07	-0.03		
6.Learning Engagement	51.56 ± 15.28	-0.12**	0.21**	0.01	0.04	0.19**	
7.usage time	1.26 ± 1.13	0.07	-0.07	-0.02	0.06	0.03	0.01

* at the 0.05 level (two-tailed), ** at the 0.01 level (two-tailed), *** at the 0.001 level (two-tailed) correlation is significant

### The mediating role of agency and the moderating role of growth mindset

**Fig. 2:**
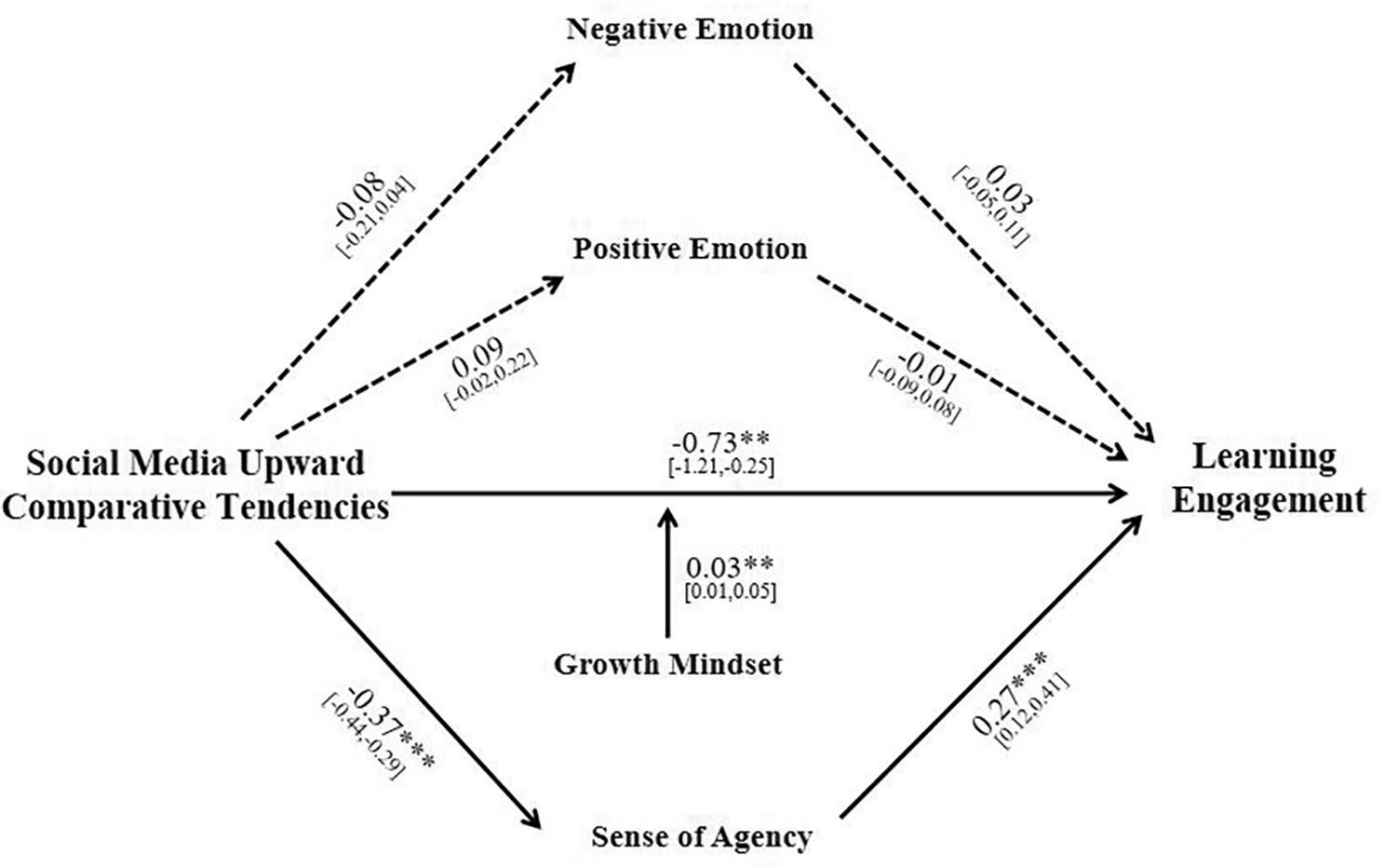
The relationship between social media upward comparative tendencies and learning engagement

After controlling for the usage time on social media applications, the SPSS 26.0 macro Process 3.5 Model 5 was employed to examine the mediating role of agency and positive-negative emotions, as well as the moderating role of growth mindset. The model (*R*^2^ = 0.09, *F* = 9.40, *p* < 0.001) are depicted in Fig. 2.

### Simple slope analysis of growth mindset

**Fig. 3:**
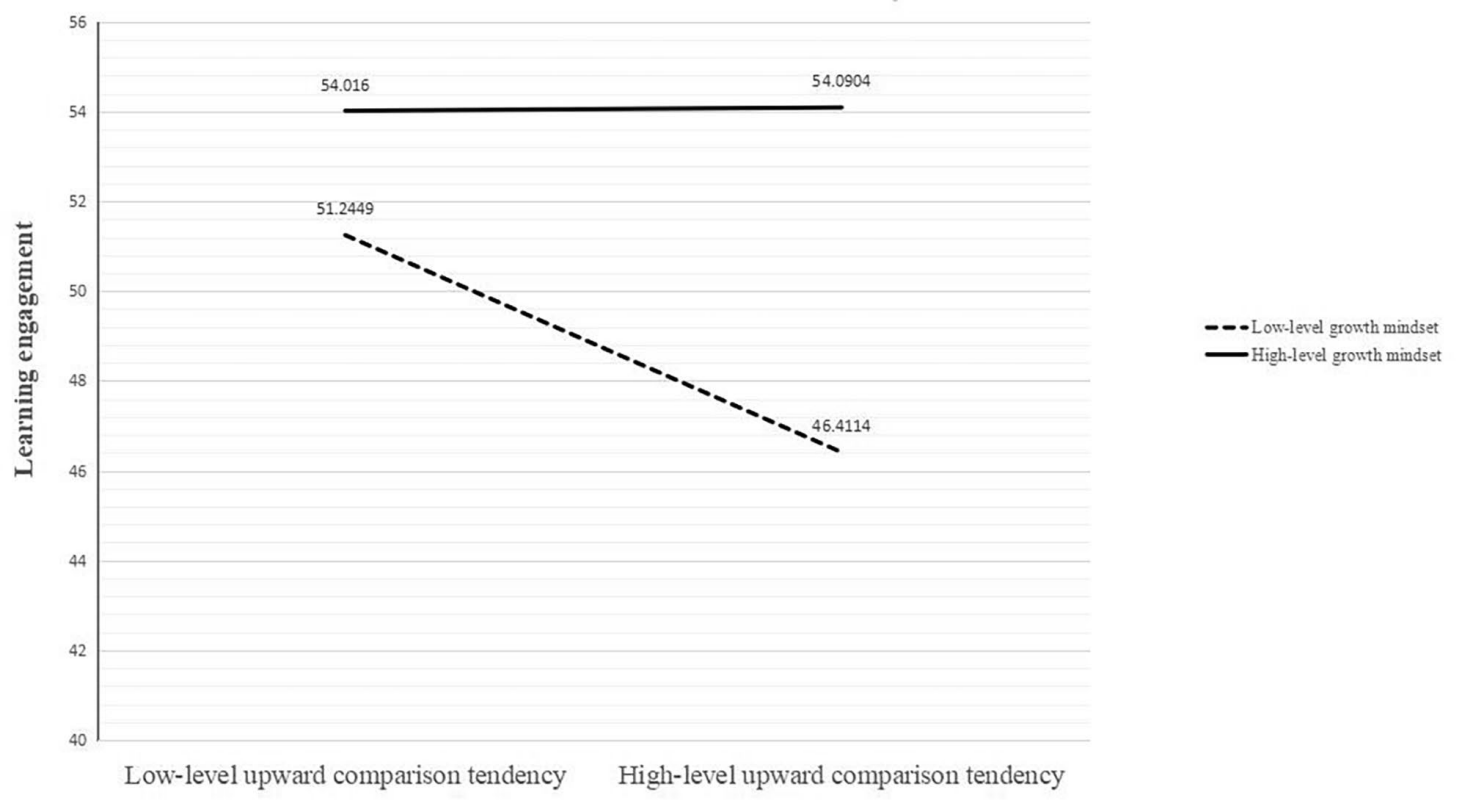
The Impact of Upward Comparison Tendencies on Learning Engagement of Adolescents with Varying Levels of Growth Mindset in the Context of Social Media

To further examine the moderating effect of growth mindset, a simple slope analysis was conducted. Figure 3 illustrates the results. Under conditions of low-level growth mindset (*M*-1*SD*), the upward comparison tendency on social media significantly and negatively predicted adolescents’ level of learning engagement (*β*=-0.26, *SE* = 0.09, *t*=-2.96, *p* = 0.003, 95% CI=[-0.44,-0.09]).

### Research 2

The research 1’s results indicates that the cognitive pathway plays a predominant role in the impact of upward social comparison tendency on learning engagement among adolescents on social media. Research 2 aims to explore the instant effects of upward social comparison on learning engagement and its underlying mechanisms. Due to the stronger emotional contagion of short videos compared to the formats of images and text, the results are more easily observed. Subsequently, there is currently a lack of experiments that use short videos as research materials. Therefore, in Research 2, short videos posted on social media will be employed as experimental stimuli for upward social comparison.

### Method

#### Participants

A total of 136 students from a public middle school in Fujian province participated in the study, including 64 boys and 72 girls (*M*(age) = 14, *SD* = 1.75). They were randomly assigned to either the upward comparison group (68 participants, 32 males, 36 females) or the control group (68 participants, 32 males, 36 females). On average, participants reported using social media for approximately 0.78 ± 0.23 h per day.

#### Experimental design, procedures and materials

The experiment utilized a single-factor between-subjects design. The independent variable was the level of social media upward comparison, and learning engagement level served as the dependent variable. The experiment took place in a quiet room where participants individually completed computer-based tasks. Initially, participants were informed that they would be rating videos as part of a project. They then proceeded to watch short videos. After watching the videos, participants evaluated their positive and negative emotions, sense of agency, willingness to engage in learning, and completed the growth mindset scale. The experiment lasted approximately 30 min, and participants were thanked for their participation and provided with a small gift at its conclusion.

#### Measure

##### Manipulation and evaluation of social media upward comparisons

Prior to the experiment, measures were taken to mitigate the potential influence of comparison fields and social distance on learning engagement. Thirty short videos (with an average duration of 1.5 min) showcasing teenagers’ learning abilities and achievements were selected from popular platforms such as Douyin, Kuaishou, and XiaoHongShu (the most popular Chinese social media App). Additionally, 45 middle school students who did not participate in the formal experiment were recruited to evaluate the direction and intensity of comparison elicited by these videos. Participants rated their own performance relative to the characters in each video on a 7 point scale ranging from − 3 (*characters in the video performing much worse*) to + 3 (*characters in the video performing much better*).

Based on these evaluations, four short videos with the highest score (*M* = 2.57, *SD* = 0.34) among the thirty were selected as Video A (duration: 5 min and 30 s), which served as material for the upward comparison group. Similarly, four short videos with the lowest score (*M*= -1.28, *SD* = 0.21) were chosen as Video B (duration: 5 min and 45 s), representing material for the control group. At the start of the formal experiment, both groups watched Video A and Video B respectively. After viewing each video, participants were asked to compare their own performance to characters of short video using a 7-point scale (-3 to + 3). Higher scores indicated a greater degree of upward social media comparison.

##### Positive - Negative emotions

The positive-negative emotion assessment method adopted for this study was adapted from Diener and colleagues [66]. After watching the video, participants were asked to rate their current emotional state using three positive words *(happy, cheerful, hopeful*) and three negative words (*sad, frustrated, hopeless)*. Participants rated each word on a scale ranging from 0 (*no feeling)* to 7 (*feeling very strongly*). Higher scores indicated a greater intensity of the corresponding emotional experience.

##### Sense of agency

To assess participants’ sense of agency in relation to their current learning environment, the determination-sense of agency assessment questionnaire developed by Lerner and Keltner [67] was utilized as a reference. Participants were asked to indicate how controllable they felt their learning life was in the given situation. A rating scale from 1 (*completely uncontrollable*) to 7 (*completely controllable*) was used.

##### Learning engagement

The situational projection questionnaire adapted from Li and colleagues [68], for measuring participants’ willingness to engage in learning, with higher scores indicating greater commitment to learning.(See *Measurement of Learning Engagement* in Appendix).

##### Growth mindset

The measurement of growth mindset in Research 2 followed the same method as Research 1.

## Results

### Validity test of social media upward comparison manipulation

After excluding three participants with identical ratings, an independent samples t-test was conducted to assess the difference in comparison scores between the upward comparison group and the control group. The results revealed a significant difference (*t* = 13.46, *df* = 131, *p* < 0.001), indicating that the upward comparison group (*M* = 2.14, *SD* = 0.97) exhibited a significantly higher level of comparison than the control group (*M*=-0.73, *SD* = 1.46). These findings confirm the successful manipulation of social media upward comparison. See Table 2 for Research 2 descriptive statistics and relevant analysis results.

**Table 2:** Descriptive statistics and correlation coefficient matrix of each variable in Study 2(*n* = 133)

	M ± SD	1	2	3	4	5
1.Instant Social Media Upward Comparison	0.76 ± 1.89					
2.Sense of Agency	5.44 ± 1.39	0.09				
3.Positive Emotion	6.27 ± 3.17	0.39**	0.21*			
4.Negative Emotion	6.35 ± 4.19	-0.52**	-0.03	-0.62**		
5.Learning Engagement	2.93 ± 1.61	-0.10	0.10	0.42**	-0.39**	
6.Growth Mind	21.09 ± 7.11	-0.13	0.11	0.34**	-0.17*	0.67**

### Multiple mediating effects and moderating effects of growth mindset

**Fig. 4:**
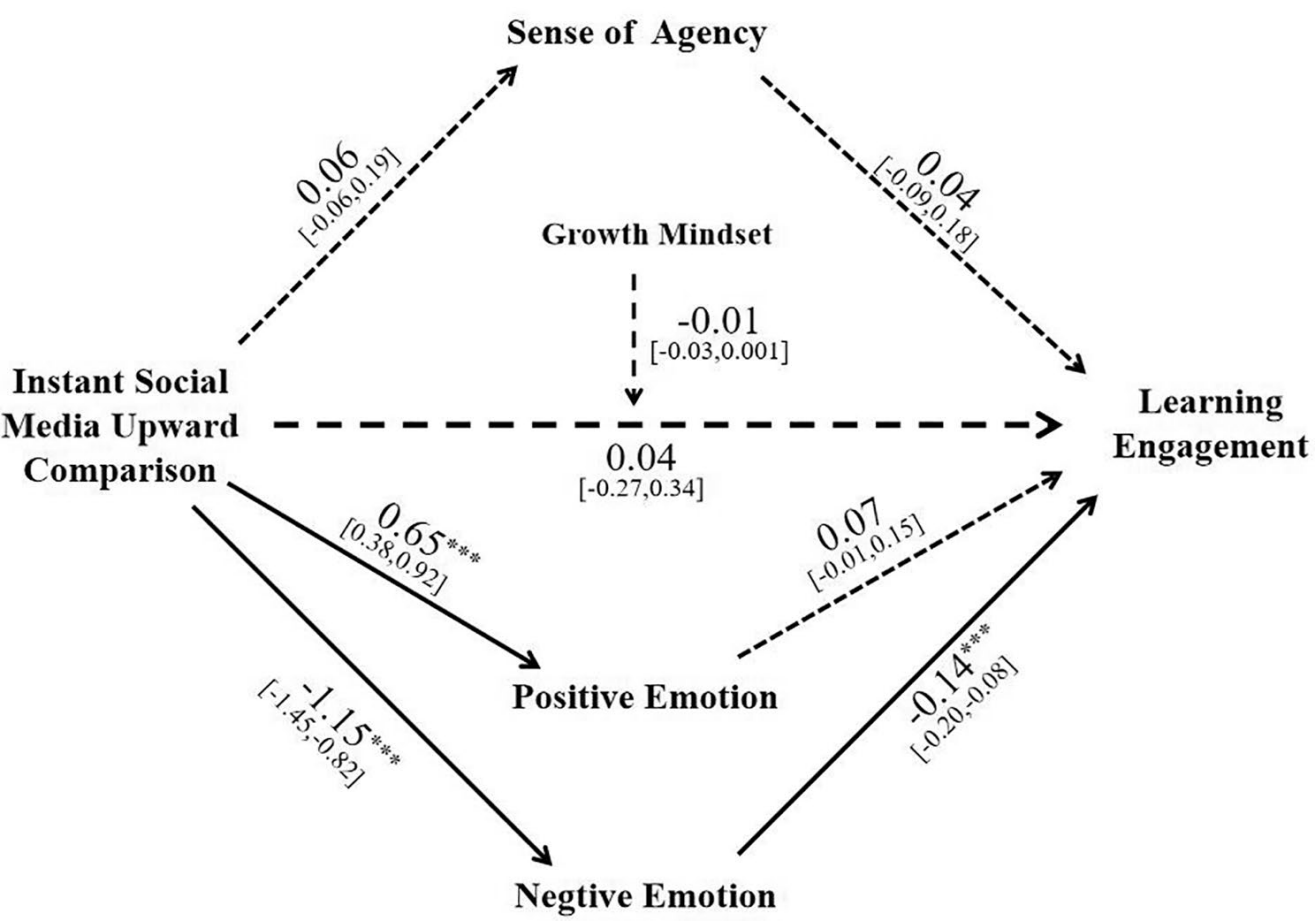
The relationship between Instant social media upward comparison and learning engagement

To investigate the mediating role of sense of agency and positive-negative emotional experience, as well as the moderating role of growth mindset, the SPSS 26.0 macro program Process 3.5 Model 5 was employed. The model( *R*^2^ = 0.59, *F* = 29.86, *p* < 0.001) depicted in Fig. 4.

### General discussion

#### The relationship between social media upward comparison and learning engagement

This study examines the effects of social media upward comparison tendencies and instant upward comparison on adolescents’ learning engagement through two researches, providing a comprehensive perspective on understanding upward comparison on social media. These findings support H1and H2. The Research 1’s result revealed that the upward comparative tendencies on social media significantly negatively predicted the level of learning engagement. This finding expands the applicability of social comparison theory [22] by demonstrating that even in virtual social environments, an upward comparative tendency can reduce the level of learning engagement. Additionally, it extends the developmental sociocultural interaction model of adolescents’ learning engagement [69], suggesting that the individual behavioral trait of upward comparative tendency on social media can also significantly predict lower levels of learning engagement. According to Self Determination Theory (SDT), individuals’ self-concept stability determines their expectations and internal motivation for their own behaviors [70]. The upward tendency on social media leads to an instability in individual self-concept construction [71], which weakens adolescents’ internal motivation for learning and positive expectations for learning outcomes, resulting in decreased learning engagement.

The results of Research 2 indicated that instant social media upward comparison had no direct significant effect on adolescents’ willingness to engage in learning.This result is inconsistent with previous research [35], which may be due to differences in participants and the individual definitions of constructive outcomes. The subjects of study were young males [35], while the current study focuses on adolescents. Secondly, it is considered that Chinese adolescents generally value academic outcomes, which may be due to influences from parental attitudes and school values, suggesting that some adolescents may not perceive learning as a form of constructive outcome. This also reflects the impact of Chinese social culture on adolescent behavioral intentions.This finding does not support cognitive self-regulation strategies [38], possibly because a visual presentation of a single learning outcome does not enhance mental contrasts clarity [72]. Therefore, it does not directly promote adolescents’ intention to engage in learning. This suggests that presenting different positive outcomes is helpful for increasing the degree of psychological contrast. Furthermore, we speculate that social media upward comparison may have a “cumulative effect,” and its directly impact on learning engagement may only be observed when instantaneous social media upward comparison reaches a certain degree. Instead, it is the comparative tendency trait that primarily influences learning engagement.

### Cognitive path

#### The mediating role of sense of agency

The upward comparison behavior tendencies was found to have a significant negative impact on the Sense of Agency, while the Sense of Agency had a significant positive impact on learning engagement. These findings support Hypothesis 3. The conclusion highlights the crucial role of sense of agency in individual goal motivation. Influenced by Chinese traditional culture and family social education values, compared to adolescents in Western cultures, Chinese adolescents link academic outcomes and the future more closely and view current academic performance as having a significant predictive effect on the quality of life in the future. Therefore, Chinese adolescents with a high sense of agency believe that their learning behaviors will yield higher academic achievements and benefit their present and future lives. This belief is reflected in their increased level of learning engagement. However, the disparity arising from upward comparison reduces the expectations adolescents have for their own future, weakening the connection between learning and the future. Drawing from learned helplessness theory [43], it can be inferred that the decrease in self-efficacy resulting from upward comparison leads to a sense of helplessness, wherein individuals believe that no matter how hard they try, they cannot improve. This further manifests as a decrease in learning input.

However, in Research 2, it was observed that the mediating effect of the Sense of Agency was not significant, and instantaneous social media upward comparison did not weaken adolescents’ Sense of Agency. This finding suggests two important points. Firstly, it indicates that cognitive changes induced by social media upward comparison occur at a slower pace than emotional changes. Secondly, it suggests that cognitive changes resulting from social media upward comparison have a temporal aspect. According to reinforcement learning theory, the development of a sense of agency requires individuals to learn the relationship between their own behavioral responses and environmental outcomes, which is based on multiple learning processes [73]. However, instant upward comparison does not meet this condition, thus it does not lead to changes in the sense of agency. Furthermore, These results suggest that educators should strive to create an interactive model of positive feedback in daily learning and life. They should also help adolescents establish a causal link between their own efforts and positive outcomes through positive interactions. They underscore the importance of fostering sense of agency and establishing positive feedback loops to enhance students’ motivation and engagement in learning.

### Affective path

#### The mediating role of positive emotions

The findings from Research 1 suggests that the upward comparative tendency of social media does not lead to significant changes in emotions. This finding is consistent with previous studies [74]. However, in the results of Experiment 2, the mediating effect of positive emotions was also not significant. Although upward comparisons presented in the form of short videos on social media can better bring pleasure to adolescents, this positive emotional experience does not significantly increase the willingness to engage in learning. They are also different with previous research [35], which indicates that positive emotions can stimulate exercise intentions, but the increase in positive emotions in this study did not increase adolescents’ willingness to learn. This may be due to the fact that positive emotions increase HRV, a state that is more akin to readiness for physical activity, and there is immediate feedback after exercise (secretion of dopamine, serotonin). However, such immediate feedback is harder to come by after studying. Therefore, the observed effect of positive emotions on the willingness to learn was not significant in Experiment 2. In addition, previous research has mainly shown that positive emotions promote engagement in learning by enhancing self-efficacy. But upward comparisons could potentially trigger a reduction in self-efficacy [25], thereby offsetting the positive impact of positive emotions on the willingness to engage in learning.

#### The mediating role of negative emotions

In Research 1, no significant changes in negative emotions due to an inclination toward upward comparison were found, thus verifying the previous hypothesis that emotional changes primarily exist in the immediate state of upward comparison. It also continues to prove that the speed of emotional changes differs from that of cognition. In Research 2, the mediating role of negative emotions was significant between upward comparison and the willingness to engage in learning. This is completely opposite to the direction of emotion elicited in previous study [36]. This may be due to the fact that in this study they recalled viewing content from their regularly followed Instagram bloggers, where the aspects of comparison are more about material conditions and physical appearance, which, compared to learning outcomes, are harder to change through personal effort. Therefore, it presented a situation where positive emotions decreased while negative emotions increased. The differences between the two studies shows that the complexity of the “behavior-result” change may be key to whether upward comparison can stimulate positive emotions, eliminate negative emotions, and promote constructive behavior. By viewing the excellent academic achievements of their peers, Chinese adolescents were able to dispel the uncertainty about the results of their own efforts in studying, thus enabling them to engage in learning more effectively. In contrast, watching others display superiority in material conditions and physical appearance does not inspire individuals to envision a future, and improving one’s own material conditions and appearance is hard to achieve simply through personal efforts. Therefore, future research may further focus on the role that the difficulty of changes in “behavior-result” plays in upward comparisons.

Besides, decreasing negative emotions generated from browsing content related to upward comparison can enhance current personal resources and promote adolescents’ willingness to engage in learning. This also elucidates the role model power in promoting constructive behavior at the emotional level.

Worthily,the disparity between the findings of Research 1 and Research 2 suggests that the rate of emotional contagion on social media may be transient. it indicates that in instant social media upward comparison, individual affective states change at a faster pace than cognitive states. And the speed of emotional change is faster than the speed of cognitive change. Further investigation is warranted to determine whether the improvement in learning engagement induced by instant social media upward comparison is sustainable.In summary, these results highlight the complex interplay between emotions and social media upward comparison on adolescents’ learning engagement.

#### The moderating effect of growth mindset

This study also revealed that growth mindset only plays a significant moderating role between the tendency for upward comparison behavior on social media and learning engagement, but does not have a significant moderating effect on instant upward comparison on social media. This finding partially supports hypothesis 5. These results are consistent with previous findings regarding the promotion of learning through growth mindset [56]. While the results of this study also indicate the moderating role of growth mindset in upward comparison, the direction of this moderation differs slightly from previous research findings [52]. The study found that among adolescents, a high growth mindset does not act as a factor in stimulating constructive behavior, but rather displays a protective role. Adolescents with a high growth mindset can maintain a relatively high level of engagement in learning when faced with the pressure of upward comparison, whereas those with a low growth mindset show a significant decline in learning engagement under the pressure of upward comparison. This might be due to the fact that the subjects of Song’s research [52] were adult employees, who, compared to adolescents, have a stronger capacity to cope with stress. Therefore, we speculate that while a growth mindset promotes constructive behavior in individuals, it also has a protective effect, and that this protective effect may precede its promotional role since adolescents have a weaker ability to cope with stress.

Compared to adult employees, adolescents have a weaker ability to cope with stress. Based on cognitive model of stress [60], the upward tendency of social media is more likely to make teenagers perceive pressure, therefor the protective effect of growth mindset becomes prominent. Individuals with a growth mindset tend to have a future-oriented time frame [58, 75]. Despite their current unsatisfactory state, they believe that their intelligence and abilities can be developed through effort [54]. This mode of mindset provides individuals experiencing upward comparative pressure with coping resources from a future time perspective to maintain their self-concept and sense of agency, thereby attenuating the impact of social media’s upward comparative tendency on learning engagement. These findings suggest that in future quality education processes, it is crucial to prioritize the cultivation and training of growth mindset. Doing so is not only beneficial for improving students’ learning motivation but also provides them with resilience when coping with stressful events.

However, in Research 2, the moderating effect of a growth mindset was not significant. Considering the results of the emotional changes in Research 2, watching short videos of excellent peers demonstrating their learning achievements does not create stress for them. Therefore, the role of a growth mindset when individuals face stress was not demonstrated.

Finally, the results of this study also explain a phenomenon indirectly, which is that sometimes when educators hope to use role models to inspire adolescents, it could turn out a counteractive role. Instead of igniting the youths’ desire to learn, it can also create unwanted comparative stress. The findings suggest that role models intended to stimulate adolescents’ willingness to learn should possess characteristics of immediacy and strong emotional appeal. The long-term lecturing commonly used by Chinese parents and teachers not only fails to stimulate teenagers’ desire to learn but can sometimes have the opposite effect.

#### Limitations and future directions

This study has a few limitations that should be acknowledged. Firstly, the material used for instantaneous upward comparison in this study only included short videos published on social media, and the influence of text and pictures as materials was not investigated. Future research could explore the effects of different types of media content on upward comparison behavior. Besides, this experiment solely relied on self-report measures to assess the apparent sense of agency. It would be valuable for future research to incorporate implicit measures to provide a more comprehensive understanding of individuals’ sense of agency. In light of these limitations, there are several promising avenues for further investigation. Future studies could examine how different forms of media content (such as text and pictures) impact upward comparison tendencies and learning engagement among adolescents. Overall, addressing these limitations and pursuing these future directions will contribute to a more comprehensive understanding of the cognitive and emotional processes underlying social media upward comparison and its impact on adolescents’ learning engagement.

## Conclusions and implications

The findings of this study demonstrate that the upward comparative tendency on social media significantly predicts lower levels of learning engagement among adolescents. The sense of agency and growth mindset act as important psychological mechanisms between them. Besides, when adolescents engage in instant comparisons with others on social media, it reduces negative emotions, which then improve their motivation and willingness to participate in learning activities. These findings underscore the importance of fostering positive online environments for adolescents, where they can develop a healthy self-concept, cultivate a sense of agency, embrace growth mindset, and minimize negative emotional experiences associated with social media upward comparison. By promoting these factors, educators and policymakers can enhance adolescents’ learning engagement both within virtual spaces and traditional educational settings.

### Electronic supplementary material

Below is the link to the electronic supplementary material.

Supplementary Material 1

## Data Availability

The raw data supporting the conclusions of this article will be made available by the authors, without undue reservation.

## Abbreviations

CNNIC: China Internet Network Information Center
PANAS: Positive-Negative Emotion Scale
SDT: Self Determination Theory
