# Supplementary material for: The effect of social media upward comparison on Chinese adolescent learning engagement: a moderated multiple mediation model
Source: BMC Psychol. 2024 Mar 4;12:122. doi: 10.1186/s40359-024-01621-z (PMC10913617; doi:10.1186/s40359-024-01621-z)
Supplement: Supplementary file 1 — Supplementary Material 1 [file 40359_2024_1621_MOESM1_ESM.docx]

**Measurement of Learning Engagement.**

"Today is Friday evening after school. Li Wei walks on campus under the warm afternoon sun and gentle breeze with a scent of grass in the air. Some students are playing ball games on the playground while others are discussing problems in classrooms. If you were Li Wei, how many hours would you spend studying and doing homework tomorrow, Saturday? Please select a number between 0 and 7."

| None | 1hour | 2hours | 3hours | 4hours | 5hours | 6hours | 7hours |
| --- | --- | --- | --- | --- | --- | --- | --- |
| 0 | 1 | 2 | 3 | 4 | 5 | 6 | 7 |
